# Unveiling the Human Gastrointestinal Tract Microbiome: The Past, Present, and Future of Metagenomics

**DOI:** 10.3390/biomedicines11030827

**Published:** 2023-03-09

**Authors:** Konstantina Athanasopoulou, Panagiotis G. Adamopoulos, Andreas Scorilas

**Affiliations:** Department of Biochemistry and Molecular Biology, Faculty of Biology, National and Kapodistrian University of Athens, 15701 Athens, Greece

**Keywords:** gut microbiome, human gastrointestinal tract, oral microbiome, metagenomics, amplicon sequencing, 16S rRNA sequencing, metatranscriptomics

## Abstract

Over 10^14^ symbiotic microorganisms are present in a healthy human body and are responsible for the synthesis of vital vitamins and amino acids, mediating cellular pathways and supporting immunity. However, the deregulation of microbial dynamics can provoke diverse human diseases such as diabetes, human cancers, cardiovascular diseases, and neurological disorders. The human gastrointestinal tract constitutes a hospitable environment in which a plethora of microbes, including diverse species of archaea, bacteria, fungi, and microeukaryotes as well as viruses, inhabit. In particular, the gut microbiome is the largest microbiome community in the human body and has drawn for decades the attention of scientists for its significance in medical microbiology. Revolutions in sequencing techniques, including 16S rRNA and ITS amplicon sequencing and whole genome sequencing, facilitate the detection of microbiomes and have opened new vistas in the study of human microbiota. Especially, the flourishing fields of metagenomics and metatranscriptomics aim to detect all genomes and transcriptomes that are retrieved from environmental and human samples. The present review highlights the complexity of the gastrointestinal tract microbiome and deciphers its implication not only in cellular homeostasis but also in human diseases. Finally, a thorough description of the widely used microbiome detection methods is discussed.

## 1. Introduction

Recent revolutions in sequencing techniques have transformed traditional microbiology into modern microbiology. To date, microbiology constitutes a flourishing biological field that involves the study of both microbiome and microbiota. To begin with, although these terms are regularly used interchangeably, microbiome, as it was first defined in the late 1980s, is a microbial community that includes the microorganisms that cohabit in a well-defined microenvironment, whereas the living members of the microbiome that can be observed microscopically are known as the microbiota [1,2]. However, breakthroughs in molecular microbiology have given birth to the study of metagenomics which comprises all genomes and genetic products that are harbored in living samples, incorporating humans, or retrieved from environmental samples, such as water and soil, whereas metatransriptomics refers to the study of transcriptomes of microorganisms [3].

Over 10^14^ symbiotic microorganisms are living in the human body and constitute the human microbiota. Multiple projects aim to decipher all the microbiome communities that are present in the human body, and most of them are focused on the gut microbiome, which constitutes the largest group of habitants [4]. Microbiome studies support that gut inhabitants are physiological endogenous factors that produce vital vitamins and amino acids, which cannot be synthesized by the organism, and mediate cellular mechanisms, thus influencing the immune system and health [5,6]. The composition of the gut microbiome is critical for maintaining homeostasis, and the deregulation of microbial dynamics can provoke diverse human diseases including diabetes, human cancers, allergic diseases, and neurological disorders [7,8,9,10]. Nowadays, microbial studies aim to characterize all the organisms that are harbored in the human body by identifying their DNA sequences. Notably, studies have been focused on the detection of marker genes, such as the 16S rRNA gene, which is conserved in bacteria and archaea, utilizing the amplicon sequencing methodology [4]. On the contrary, the newly introduced metagenomics approach is based on whole genome sequencing techniques, hence identifying all microbial genomes that are retrieved from a sample and aims both to classify the microorganisms and reveal functional information about their contribution to human homeostasis. A plethora of microbiome projects, incorporating the Human Microbiome Project (HMP), the Integrative Human Microbiome Project (iHMP), and the European MetaHIT, have been launched worldwide in order to both decipher the human microbiome and understand the impact of these symbionts in human health and disease [11,12,13].

In the present review, we focused on deciphering the complexity of the human gastrointestinal tract microbiome that plays a critical role in homeostasis and interplays between inflammation, disease, and cancer. Moreover, the current review aims to provide a thorough description of the detection methods that are widely used for the characterization of the microbiome in the era of modern microbiology. Notably, the classic culturing techniques for identifying microbes, namely culturome, and the newest amplicon-based sequencing methods and culture-free metagenomic sequencing approaches are sufficiently depicted. Finally, the contribution of the newly introduced metatranscriptomics sequencing is also highlighted.

## 2. The Role of the Gastrointestinal Tract Microbiota in Human Health and Disease

The human body constitutes a natural habitat for microbial communities, such as diverse species of Archaea, Bacteria, Fungi, and microeukaryotes as well as Viruses, which have been detected in multiple anatomical body sites and tissues including the skin surface, the respiratory tract, the gastrointestinal or alimentary tract, the mammary gland, and the urogenital tract [14]. Notably, the core microbiome is comprised of predominant aerobe microorganisms that inhabit the skin, the nasal cavity, and the respiratory tract, whereas in the gastrointestinal tract, the anaerobes dominate (Table 1) [14]. More precisely, the alimentary tract is made up of various organs that swallow, digest and absorb food, which are the oral cavity, pharynx, esophagus, stomach, small and large intestine, and accessory organs, and is a unique environment that harbors plenty microorganisms [15].

**Figure 1:**
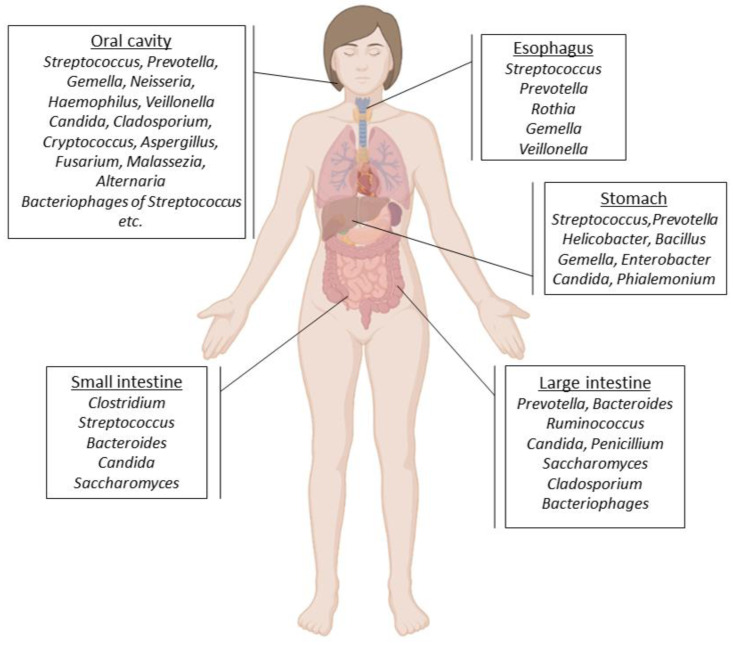
Microbes that inhabit the oral cavity [29], esophagus [30], stomach [31] as well as small and large intestines [32,33,34] in humans.

To begin with, commensal bacteria are dominant in the oral cavity. Especially, more than 1000 species have been detected and studies have shown that they mediate cellular processes and maintain homeostasis [16,17]. It is worth mentioning that Actinobacteria, Bacteroidetes, Firmicutes, Proteobacteria, Streptococcus, Spirochaetes, Synergistetes, and Tenericutes are representative phyla that have been found in this highly complex bacteria community (Figure 1). More precisely, streptococci constitute the first bacteria inhabitants of the oral cavity and under physiological conditions; they are responsible for the generation of acids by catalyzing the metabolism of carbohydrates [18]. Both *Streptococcus salivarius* and *Streptococcus gordonii* produce great amounts of alkali; hence, they contribute to human homeostasis by regulating the levels of acids in the oral cavity. On the contrary, the levels of *Streptococcus mutans* and *Porphyromonas gingivalis* are influenced by carbonic anhydrases, pH, and ions in the oral cavity; hence, they are implicated in diseases such as dental caries and periodontitis [19].

The class of Archaea is restricted since only some species of methanogens have been found, containing *Methanobrevibacter oralis*, *Methanobacterium curvum,* and *Methanosarcina mazeii* [20,21]. Various studies support that methanogens facilitate hydrogen transfer, and thus influence the growth of bacteria that are responsible for periodontal diseases. Consequently, increased levels of methanogens such as *Methanobrevibacter oralis* are related to periodontitis [20]. As for Fungi, *Candida* is in abundance in the oral cavity, whereas various species of the *Aspergillus*, *Cladosporium*, *Cryptococcus*, *Aureobasidium*, *Saccharomycetales*, and *Fusarium* genera are also present [21]. Adaptive immune responses and the host’s innate reflexes allow the cohabitation of *Candida albicans* and other microorganisms in the oral cavity [22]. These symbiotic relationships between the host and the microorganisms restrict the colonization and the growth of pathogens and promote homeostasis. However, disruptions of the physiological parameters such as temperature, nutrients, and pH that contribute to the establishment of these resident microbes, influence human pathophysiology [22]. The commensal oral microbiota colonizes all surfaces of the mouth leaving little space for pathogenic invaders, thus protecting the cavity and maintaining systemic health [23,24]. For instance, the health-associated Bacteria *Streptococcus salivarius* produces the toxin bacteriocin which prevents the growth and activity of Gram-negative bacterial species that cause periodontitis and halitosis [25]. Additionally, other *Streptococcus* species have been related to type 1 diabetes [26]. Chronic kidney diseases are also influenced by oral microbiota. In patients with chronic kidney disease, higher levels of *Candida albicans* and *Porphyromonas gingivalis* are responsible for chronic periodontitis [27]. On the contrary, the oral virome contains different types of viruses, including Human Papilloma Virus (HPV), hepatitis, and mumps viruses, as well as *Herpes simplex* and *Rabies lyssavirus*, which all have been found in saliva and are usually disease-associated. For instance, *Herpes simplex* is responsible for gingivostomatitis, whereas HPV causes several oral conditions such as focal epithelial hyperplasia, oral papillomatosis or even neck squamous cell carcinoma [28].

Although the dynamic of microbial colonies enhances oral homeostasis, a range of microorganisms has also been related to oral diseases such as dental caries, periodontitis, and cancer (Table 2). Notably, numerous pathological alterations that occur within the microbial environment can affect bacterial growth and activity, hence initiating disease. More precisely, reductions in the pH of the saliva and the increase in the lactic acid that is produced by oral bacteria such as *Streptococcus mutans*, *Bifidobacterium*, *Propionibacterium,* and *Lactobacillus* lead to dental caries [35]. In the case of periodontitis, a periodontal pocket is formed due to a gap between the teeth and the gingivae. The periodontal pocket is colonized with various bacteria species, including *Porphyromonas gingivalis*, *Porphyromonas endodontalis*, *Treponema denticola*, *Anaeroglobus geminatus*, *Eubacterium saphenum,* and *Prevotella denticola* and finally, the tissue is damaged [36]. The oral microbiome can also cause infections at different body sites leading to serious diseases. For example, clinical studies in patients with cystic fibrosis have shown that oral bacterial species have been found in the lung [37].

The human pharyngeal microbiome comprises the phyla Actinobacteria, Firmicutes, Bacteroidetes, Proteobacteria, and Fusobacteria, among which Bacteroidetes is the most abundant, while *Prevotella*, *Neisseria*, *Streptococcus*, *Campylobacter,* and *Haemophilus* are the most prevalent genera [38]. More precisely, the gram-positive *Streptococcus pyogenes* as well as *Prevotella melaninogenica* are responsible for pharyngitis, while pharyngeal colonization by Neisseria species such as *N. meningitidis* and *N. gonorrhoeae* have been detected in patients with gonococcal infections [39]. Moreover, although the pharyngeal cavity harbors a variety of pathogenic species such as *Haemophilus influenza*, *Staphylococcus aureus*, *Streptococcus pneumonia,* and *Mycoplasma pneumonia*, in many cases these residents are not attacking the immune system and the host is characterized as asymptomatic [38]. For instance, *Streptococcus pneumonia*, which is responsible for deathly pneumococcal diseases, is normally found in the human pharynx of healthy individuals but it can migrate to different tissues and cause serious infections [40]. Additionally, the human oropharyngeal virome includes a plethora of respiratory viruses such as DNA Chloroviruses [41]. For example, Chlorovirus *Acanthocystis turfacea chlorella virus 1* (ATCV-1) inhabits human mucosal surfaces such as the pharynx and produces a variety of enzymes that either enhance or impair the host’s immunity [41].

In the same manner, the esophageal microbiome is similar to the pharyngeal microbiome and is comprised of six bacteria phyla among which Firmicutes is overexpressed. Accordingly, Bacteroidetes, Actinobacteria, Fusobacteria Proteobacteria, and TM7 are also present (Figure 1). *Streptococcus* constitutes a highly abundant genus, but *Prevotella* and *Veillonella* are also in great abundance [42]. Changes in the esophageal microbiome levels can cause a plethora of esophageal-related diseases such as Barrett’s esophagus, esophageal adenocarcinoma, and eosinophilic esophagitis. For instance, the progression of esophageal adenocarcinoma can be induced by rare and abundant phage communities [43]. Recent studies support that significant variations in the abundance of microorganisms, including *Streptococcus, Prevotella,* and *Treponema*, have been detected in esophageal carcinoma tissues indicating that they constitute markers for the prognosis and diagnosis of esophageal squamous cell carcinoma [44]. Additionally, fungal esophagitis is an infection that is caused by *Candida* species or Filamentous Fungi [45].

For many years scientists believed that the stomach is a sterile organ, hence it cannot harbor any bacterial community. However, this dogma was demolished in 1982, when *Helicobacter pylori* was detected. Of note, *Helicobacter pylori* is a key player in gastric homeostasis, since it modulates the acid levels in the stomach, hence affecting the gastric microbiome. *Helicobacter pylori* infection can cause serious diseases such as chronic gastritis and carcinogenesis [31]. In gastric mucosa, the phylum of Firmicutes dominates, while Proteobacteria, Actinobacteria, Bacteroidetes, and Fusobacteria are also present [46]. Moreover, the gastric microbiota is also comprised of additional acid-resistant bacterial strains that either are grown into the stomach, or migrate from the oral cavity. Namely, these bacteria species are *Streptococcus*, *Neisseria*, *Veillonella*, *Clostridium,* and *Lactobacillus* [31].

**Table 2:** Human diseases that have been related to imbalance of the normal gastrointestinal tract microbiome.

Human Disease	Related Microorganisms	Reference
Atopic dermatitis	*Staphylococcucus aureus, Cutibacterium*, *Streptococcus*, *Acinetobacter, Gemella*	[47,48]
Cystic fibrosis	*Streptococcus* species	[49]
Depression	*Coprococcus, Sellimonas, Clostridium, Hungatella*	[50,51]
Autism	*Clostridium bolteae*	[52]
Asthma	*Clostridia, Proteobacteria*	[53,54]
Obesity	*Actinobacteria, Bacteroidetes*	[55,56]
Tuberculosis	*Mycobacterium tuberculosis, Bacteroides fragilis, Prevotella, Enterococcus*	[57]
Periodontal diseases	*Spirochaetes*, *Synergistetes, Bacteroidetes*	[58]
Dental caries	*Streptococcus mutans*, *Lactobacillus* spp., *Candida albicans*	[35]
Oral cancer	*Streptococcus* species	[59]
Esophageal cancer	*Tannerella forsythia*, *Porphyromonas gingivalis*	[60]
Cardiovascular disease	*Campylobacter rectus*, *Porphyromonas gingivalis*, *Porphyromonas endodontalis*, *Prevotella intermedia*	[61]
Rheumatoid arthritis	*Veillonella*, *Atopobium*, *Prevotella*, *Leptotrichia*	[62,63,64]
Parkinson’s disease	*Lachnospiraceae*, *Faecalibacterium*, *Lactobacillus*, *Akkermansia*, *Bifidobacterium*	[65]
Alzheimer’s disease	*Spirochaetes*	[66]
Diabetes	*Aggregatibacter*, *Neisseria*, *Gemella*, *Selenomonas*, *Actinomyces*, *Fusobacterium*, *Streptococcus*	[67,68,69]

Especially, gut microbiota or gut microbiome is the largest microbiome community in the human body and has drawn for decades the attention of scientists for its significance in medical microbiology. Of note, the gut microbial community constitutes a dynamic and complex collection of all microorganisms that are accommodated in the gastrointestinal tract, including Bacteria, Archaea, Fungi, and Viruses [2]. Physiologically, these populations cooperate in different ways to provide immune defense against pathogenic organisms, regulate metabolic processes, and support cellular homeostasis, being characterized as the host barriers to infections [70]. More precisely, the intestinal microbiome is basically composed of multiple bacterial species (~50 bacterial phyla) in which Bacteroidetes and Firmicutes are the dominant phyla in a healthy human gut. Additionally, Proteobacteria and Actinobacteria are found in abundance, whereas the genera *Bifidobacterium*, *Escherichia*, *Clostridium,* and *Akkermansia* are also detected at lower levels [9,71]. Moreover, most studies support that the human gut archaeome includes two methanogenic classes of Archaea: the Methanobacteriales and the Methanomassiliicoccales. *Methanobrevibacter smithii* and *Methanosphaera stadtmanae* are the main representative species of Methanobacteriales, whereas in the case of Methanomassiliicoccales, *Candidatus Methanomassiliicoccus intestinalis*, *Methanomethylophilus alvus*, *Methanomassiliicoccus luminyensis* and the strains Mx02, Mx03, and Mx06 are dominant [72]. Additional studies have also reported that members of Haloarchaea, including *Haloferax* and *Halorubrum* spp., are also present in the human gut [73,74]. To continue with, fungal communities inhabit the gastrointestinal tract and support the immune system, and hence maintain cellular homeostasis [75]. The phylum of Ascomycota is overrepresented in gut mycobiome, whereas Basidiomycota and Mucoromycota exhibit lower expression levels [76,77].

Notably, a plethora of intestinal protozoan helminthic parasites that belong to micro-eukaryotes have been detected in the human gut and are responsible for various infections such as giardiasis, amoebiasis, and cryptosporidiosis [78]. More precisely, infections by *Giardia intestinalis* result in giardiasis, Cryptosporidium spp. causes cryptosporidiosis, *Entamoeba histolytica* is responsible for invasive amoebic infections, whereas infections by *Cyclospora cayetanenensis* lead to cyclosporiasis [79,80]. On the contrary, infections by helminthic parasites including *Ascaris lumbricoides*, *Ancylostoma duodenale, Trichiuris trichiuria,* and *Necator americanicus* affect human health since they disturb mental and physical growth but there is no evidence that they are deathly [81]. Finally, the gut also harbors viruses which contribute to homeostasis in human physiology. Briefly, DNA bacteriophages, such as *Caudovirales* and members of other families such as Myoviridae and Siphoviridae, dominate in the gut virome, while *Circovirus* and a small number of other eukaryotic and archaea viruses are present [71,82,83,84].

Furthermore, the high complexity of the gut microbiome contributes to the development of the immune system and gut homeostasis and elicits effective immune responses against invasive pathogens, including viruses. Microbial balance in the gut can ensure immunity by blocking invading pathogens not only in the gastrointestinal tract but also in multiple human organs. For instance, many studies support that gut microbes can eliminate infections by lung-associated viruses, such as the flu virus and SARS-CoV-2 by producing antiviral proteins [85]. This crucial role of gut microbiota depends on cellular mechanisms that regulate microbial metabolites and molecular pathways both in the host and their inhabitants [86].

The gut microbiota has been related to multiple diseases including brain disorders, neuropsychiatric disorders, cardiovascular diseases, type 2 diabetes, asthma, and human malignancies (Table 2). Firstly, recent studies support that the gut microbial community affects communication and interactions between the central and enteric nervous systems, namely the gut–brain axis [87]. Psychiatric disorders such as depression and anxiety have been connected to gut microbiome composition, since various bacteria genera, including *Coprococcus*, *Sellimonas*, *Clostridium,* and *Hungatella*, are involved in the synthesis of the key neurotransmitters serotonin, GABA, and glutamate [88]. In the same manner, increased levels of Actinobacteria, Bacteroidetes, and Protobacteria are also responsible for the emergence of depressive disorders (Table 2). Alterations in the expression levels of gut bacteria species are responsible for the progression of Parkinson’s disease. More precisely, a decrease in the levels of *Lachnospiraceae* and *Faecalibacterium* and an increase in the levels of *Lactobacillus*, *Akkermansia*, and *Bifidobacterium* have been associated with poor prognosis [65]. As far as type 2 diabetes is concerned, multiple studies have reported that the levels of Firmicutes and Clostridia are significantly lower in patients with type 2 diabetes [89]. Gut-microbiota-derived molecules such as short-chain fatty acids (SCFA), trimethylamine-N-oxide (TMAO), and uremic toxins are implicated in the development of cardiovascular diseases (Table 2). Changes in microbiota profile can lead to an increased risk of cardiovascular diseases. For instance, in adipose tissues, the abundance of *Akkermansia* has been correlated with inflammation and lipid metabolism [90], whereas obesity is affected by the lower ratio of Bacteroidetes to Firmicutes [91]. Moreover, in the case of asthma, the Proteobacteria are overexpressed, whereas the levels of Firmicutes and Bacteroidetes are decreased [92]. Finally, cancer-associated studies suggest that the human microbiome has a huge impact on carcinogenesis by influencing the proliferation of the host cells, mediating host metabolism, and affecting cellular immunity [93,94].

## 3. Detecting Microbes

Undoubtedly, identifying microbes and studying their functional role as well as their great diversity and complexity in the human body constitutes an attractive research field. However, the identification and quantification of the gut microbiome remain incomplete tasks due to its dynamic [95]. In the last decades, technological innovations in molecular microbiology have enabled the characterization of microbiomes and multiple studies have attempted to decipher the complex features of microorganisms and their role in human physiology. Up to date, four strategies for studying the human microbiome are widely used: a. the traditional culturomics approach that is based on the type of microbes, b. the amplicon-based DNA sequencing method, c. the modern whole genome metagenomics strategy, and d. the quite new metatranscriptomics approach. In this section, all the available strategies are thoroughly discussed.

### 3.1. Culturomics

More than a hundred years have passed since the first technique for identifying microorganisms was introduced. More precisely, in the early 1880s the plating method for culturing and detecting microbes, based on their biochemical features, was established by Robert Koch. Although classical microbiome studies are based on cultivation techniques, they enable the detection of only half of the gut bacteria. Over the years, improvements in culture conditions, the development of molecular technologies, and the advent of both Mass Spectrometry and Sanger sequencing have led to culturomics, a recently adapted technique that combines culture-dependent approaches and high-throughput methods for mapping the microbiome [96,97].

**Figure 2:**
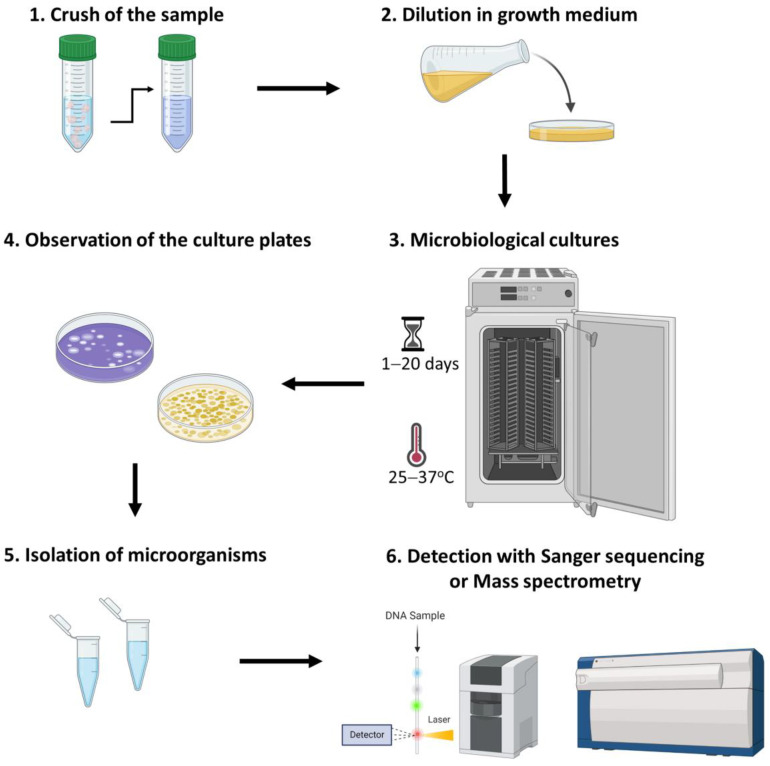
Culturomics method for the detection of microbiome.

The culturomics approach includes distinct and sufficient steps (Figure 2). Briefly, the first step involves the crushing of the living or environmental samples and their dilution in a liquid growth medium. The selection of the appropriate culture media depends on the type of microbial species that are cultured since each microorganism has different nutritional requirements (Table 3). More precisely, based on the agar concentration growth media are classified into solid, semisolid, and liquid [98]. In addition, culture plates are incubated for 1–20 days at 25–37 °C. Of note, incubating oxygen levels vary among different microbial communities since most of them are aerobic, whereas the gastrointestinal tract harbors anaerobic species [99,100,101]. In addition, culture plates are incubated for 1–20 days at 25–37 °C. Accordingly, the culture plates are observed, and different phenotypes are isolated and grown individually. For the detection of the isolated bacterial species, cultures are harvested, and extracts are purified [70,102]. The lysates can be detected by either 16S rRNA/rDNA sequencing or matrix-assisted laser desorption/ionization-time-of-flight mass spectrometry (MALDI-TOF MS) [103].

As for the advantages of culturomics approaches, they are high-throughput methods that are between the culture-dependent and the culture-independent strategies and can identify a high number of novel species that are visible in bacterial colonies. Moreover, due to the growing conditions, culturome enables the selection of the desired target and efficiently provides microbial isolates [104,105]. As for its drawbacks, culturome is a time-consuming and cost-ineffective approach that also requires accurate experimental manipulations. Furthermore, culturome results are highly influenced by the quality of culture media and environmental conditions such as temperature. Lastly, an additional limitation is that uncultured microbiota cannot be observed and hence detected (Table 4).

### 3.2. Amplicon Sequencing Analysis

Molecular biology research has been dramatically enhanced due to the introduction of high-throughput sequencing techniques that enabled a plethora of applications including molecular microbiota studies. More precisely, microbiome analysis has been radically transformed due to advances in sequencing applications including amplicon and whole genome approaches. Especially, amplicon sequencing constitutes a well-established method that includes the amplification and the detection of target genes that harbor characteristic and conserved motifs. Notably, studying microbiomes using amplicon sequencing can be divided into two approaches: a. 16S rRNA amplicon sequencing for identifying the prokaryotic sequences of Bacteria and Archaea and b. internal transcribed spacers (ITS) sequencing for the detection of the eukaryotic Fungi microcommunities. The prokaryotic 16S rRNA gene has conserved regions which are disrupted by nine variable regions that are used for the phylogenetic classification of genera in various microbes within a sample [111,112]. On the contrary, the rRNA cistron has the ITS region that is used as a DNA marker for the detection of fungal species [113].

Multiple studies support that amplicon sequencing has numerous advantages as compared to culturing methods [114]. To begin with, the 16S and the ITS rRNA amplicon sequencing method is a cost-effective technique to identify strains that may not be found using culturing methods. Moreover, amplicon sequencing is based on PCR amplification and thus requires low biomass. Additionally, next-generation sequencing (NGS) techniques enable parallel high-throughput generation of reads, the amount of the produced data is relatively small, and the bioinformatics analysis is quite simple [115]. However, PCR amplification can introduce biases such as false-positive and false-negative samples. Furthermore, amplicon sequencing cannot discriminate dead from living bacteria.

**Figure 3:**
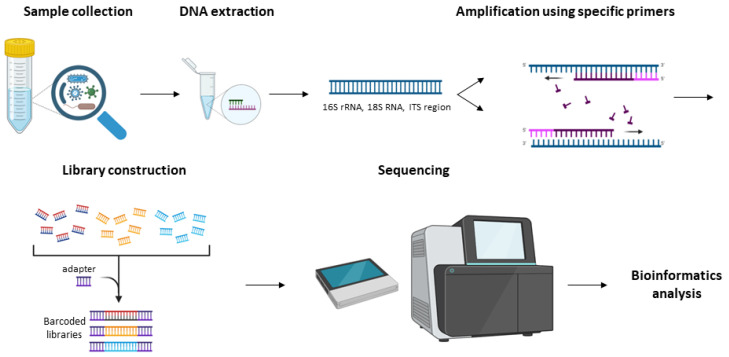
Demonstration of the workflow for the 16S rRNA and ITS amplicon sequencing.

Notably, the benefits of amplicon sequencing and its wide usage in the scientific community have led to a great number of available protocols and workflows for studying the microbiome [114,116,117]. Especially, the NGS platforms, Illumina and Ion Torrent, utilize consensus adapters that bind specifically to conserved regions for amplifying and sequencing the targets of interest [118,119,120,121]. Additionally, NGS technology enables a high quality of generated data. Both approaches share well-defined steps (Figure 3) that include the following: 1. The collection of the sample; 2. Optional culturing of the bacterial or fungal samples in plates at 37 °C for 18–72 h; 3. Isolation of unique bacterial or fungal colonies; 4. Genomic DNA extraction; 5. 16S rRNA gene or ITS amplification procedure is divided into two PCR steps for amplifying and adding the barcodes; 6. Library construction based on the sequencing platform that is used; 7. Amplicon sequencing; and 8. Bioinformatics analysis [122,123]. On the contrary, PacBio and ONT platforms perform full-length 16S sequencing and provide real-time sequencing and direct analysis of the microbiome with greater taxonomic resolution compared to NGS results [124].

### 3.3. Metagenomics

Metagenomics aims to sequence the whole DNA that is available in a particular sample/environment, providing more information compared to amplicon sequencing. This method not only detects all the species that inhabit an environment, such as the human gut, but also generates information about the genomic profile of the sample. Metagenomics can be divided into two approaches: taxonomic metagenomics and functional metagenomics. Moreover, taxonomic applications aim to investigate the phylogenetic relationships between the detected sequences and the known microorganisms. Taxonomic profiling is widely used for identifying all the microbes (rare and abundant) included in a sample [125]. On the contrary, functional metagenomics techniques are focused on the identification of functional genes and novel proteins that contribute to the microbial population’s activity. Functional metagenomics can give answers to different biological issues such as how the microbes affect the functional pathways of their hosts and how they are implicated in various pathologies [126].

**Figure 4:**
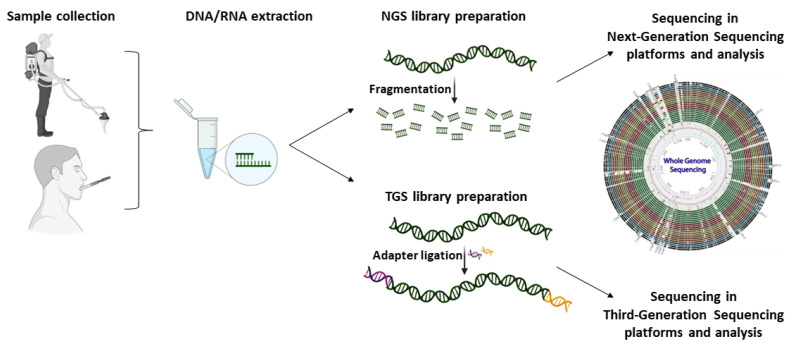
Workflow of whole genome and whole transcriptome sequencing approaches for metagenomics and metatranscriptomics studies using next- and third-generation sequencing platforms.

Both NGS and TGS approaches enable metagenomics sequencing; however, due to their chemistries, the library preparation steps, and the bioinformatics analysis procedure differ. Briefly, a metagenomics experiment is comprised of fundamental steps including the sample collection, the isolation of the DNA, the construction of either the NGS or TGS library, and, finally, the DNA sequencing (Figure 4). Of note, NGS libraries require the fragmentation of the DNA sample, whereas TGS enables the direct detection of full-length DNA sequences. In contrast to amplicon sequencing, metagenomics is more expensive and requires a greater amount of output data to perform accurate bioinformatics analysis and detect all the microorganisms that are present in a sample. Metagenomics are not only culture-independent methods but also PCR-free protocols that enable the absolute quantification of all genomes that are present in a sample without introducing PCR biases. However, the samples are often contaminated with host-derived DNA and the high complexity of the generated data can incommode the analysis (Table 4).

### 3.4. Metatranscriptomics

The newly introduced metatranscriptomics approaches enable the study of the transcriptomic profile of microorganisms and have the potential to gain deeper insights into the composition of the human microbiota. Of note, whole RNA sequencing strategies can offer the quantification of the expression levels of active genes in complex microbial communities and total mRNA sequencing unravels the expression patterns of microbes within a host, such as the human gut [127]. Moreover, metatranscriptomic data are used for the determination of transcriptionally active microbial populations that interact with hosts, including humans, and for the detection of the active metabolic pathways that are connected to human diseases [110]. In healthy individuals, metatranscriptomics approaches are used to determine the activity of microbial communities that contribute to homeostasis by producing vital vitamins and amino acids or being involved in carbohydrate metabolism [128].

In the same manner as metagenomics, metatranscriptomics includes two distinct steps: the wet lab procedures and the bioinformatics analysis. As for the wet lab step, it contains the isolation and purification of the microbiome RNA, the construction of the metatranscriptomic sequencing libraries, and, finally, the RNA seq in the NGS sequencing systems (Figure 4). The second step of metatranscriptomics analysis comprises the analysis of the generated data. Although a number of bioinformatics algorithms have been developed for the study of the metagenome, the design of computational methods for analyzing metatranscriptomic data remains a challenging process [129]. It should be mentioned that, except for the NGS platforms that have already been established in the field of microbiology for the study of metatranscriptome through RNA-seq, the brand-new direct RNA nanopore sequencing approach constitutes a highly promising strategy. In brief, the method enables the characterization of the RNA without the reverse transcription and PCR amplification steps, and performs absolute quantification of RNA, and, thus, is expected to be a milestone in modern metatranscriptomics [130]. Ultimately, metatranscriptomics has the ability to change our perception of the biological function of microbes in the human body and will undoubtedly enhance our efforts in understanding their involvement in cellular mechanisms that disrupt human homeostasis and promote carcinogenesis.

## 4. Conclusions

Overall, the human body is a host for different types of microorganisms among which bacteria are dominant. The human microbiota benefits the body by synthesizing vital vitamins and amino acids, thus stimulating the immune system. Especially, the gastrointestinal tract accommodates a plethora of microbes that contribute to cellular homeostasis. However, multiple studies report that immune deregulation is correlated with changes in gut microbiome leading to the development of various diseases. To date, different detection methods have been used to decipher the characteristics of the human microbiome and clarify the relationship between microbes and human health and disease. The selection of the detection method is based on the type of experiment and the issues that need to be addressed. Notably, the type and the quality of the samples, the cost, and the needed time for each approach should always be taken into consideration. Undoubtedly, metagenomics will provide novel insights into human microbiome and health and will enhance not only modern microbiological research but also clinical microbiology and epidemiology through the establishment of novel approaches for preventing, improving, and reversing microbiome-related diseases [70].

## Figures and Tables

**Table 1:** Common bacteria species found in human tracts and their oxygen requirements.

Bacteria	Oxygen Requirement
*Mycobacterium tuberculosis*	Obligate aerobe
*Micrococcus luteus*	
*Neisseria meningitidis*	
*Neisseria gonorrhoeae*	
*Bacteroidetes*	Obligate anaerobe
*Porphyromonas* sp.	
*Prevotella* sp.	
*Clostridium* spp.	
*Staphylococci*	Facultative anaerobes
*Gemella* sp.	
*Enterobacteriaceae*	
*Lactobacilli*	Aerotolerant anaerobes
*Campylobacter jejuni*	Microaerophile

**Table 3:** List of the growth media that are used for the culture of the most prevalent gut microbial communities.

Related Microorganisms	Culture Media
*Gram-positive Staphylococci*	Baird-Parker agar
*Streptococcus pyogenes*	Crystal Violet Blood Agar
*Gram-negative bacterial* species	Hektoen Enteric Agar
*Mycobacterium* species	Lowenstein Jensen Medium
*Gram-negative bacterial* species	MacConkey’s Agar
*Gram-positive bacterial* species	Mannitol Salt Agar
*Enterococcus* species	Potassium Tellurite Medium
*Pseudomonas aeruginosa*	Pseudosel Agar
*Neisseria gonorrhoeae*	Thayer Martin Agar
*Vibrio* species	Thiosulfate Citrate Bile Salts Sucrose Agar
*Salmonella & Shigella* species	Salmonella-Shigella Agar
*Salmonella* species	Wilson and Blair’s Agar
*Ectomycorrhizal fungi*	BAF Medium
*Ectomycorrhizal fungi*	Modified Melin-Norkrans Medium
*Certain fungi*	Sabouraud Agar

**Table 4:** Advantages and drawbacks of microbiome high-throughput detection methods.

Detection Method	Advantages	Drawbacks	Reference
Culturome	Visible colonies	Time-consuming and expensive	[106]
	Microbial isolates	Sterile environmental conditions	
	Selection of the target	Laborious	
Amplicon sequencing	Cost-effective	PCR biases	[107,108]
	Easy and quick analysis	False-positive and false-negative samples	
	Selection of the target	No discrimination between dead and live microbes	
	Low-biomass requirement	Limited genus-level taxonomic resolution	
Metagenomics	Culture-independent	Expensive	[109]
	Species or strain level taxonomic resolution	Complex and time-consuming analysis	
	Captures all genomes present in a sample	No discrimination between dead and live microbes	
	Culture-independent	Host-derived contamination	
Metatranscriptomics	Transcript level resolution	Expensive	[110]
	Assessment of gene expression	Complex and time-consuming analysis	
	Discrimination between dead and live microbes	Snapshot of protein expression levels Host RNA contamination	

## Data Availability

No new data were created or analyzed in this study. Data sharing is not applicable to this article.
